# Supplementary figures and images for: Single-cell profiling of lncRNAs in human germ cells and molecular analysis reveals transcriptional regulation of LNC1845 on LHX8
Source: eLife. 2023 Jan 5;12:e78421. doi: 10.7554/eLife.78421 (PMC9859043; doi:10.7554/eLife.78421)

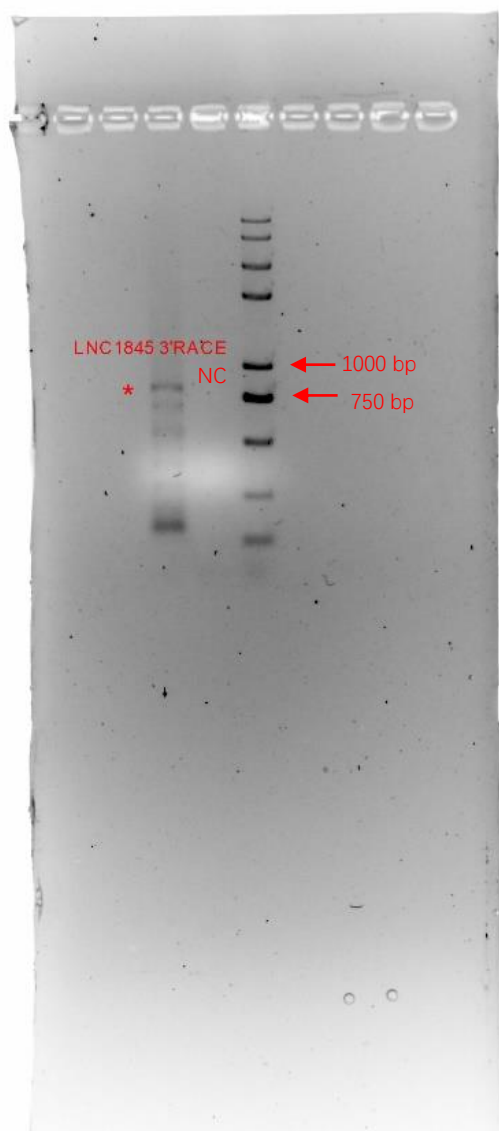

Figure S4D

Supplement: Figure 4—figure supplement 1—source data 1. [file elife-78421-fig4-figsupp1-data1.zip › Figure 4-figure supplement 1D-source data.pdf]

LNC1845

PC

NC

Marker

Figure S4B

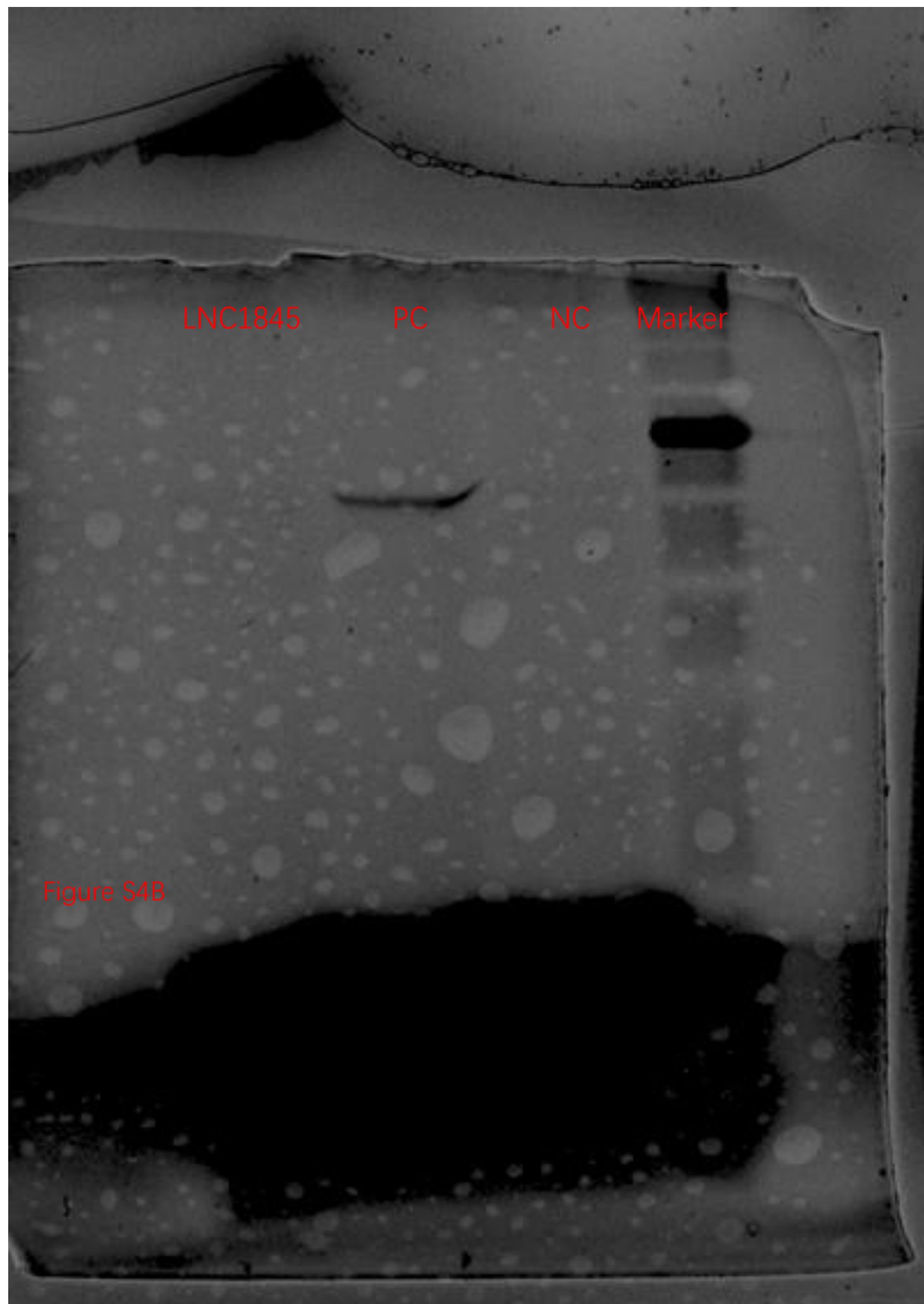

Supplement: Figure 4—figure supplement 1—source data 1. [file elife-78421-fig4-figsupp1-data1.zip › Figure 4-figure supplement 1B-source data.pdf]

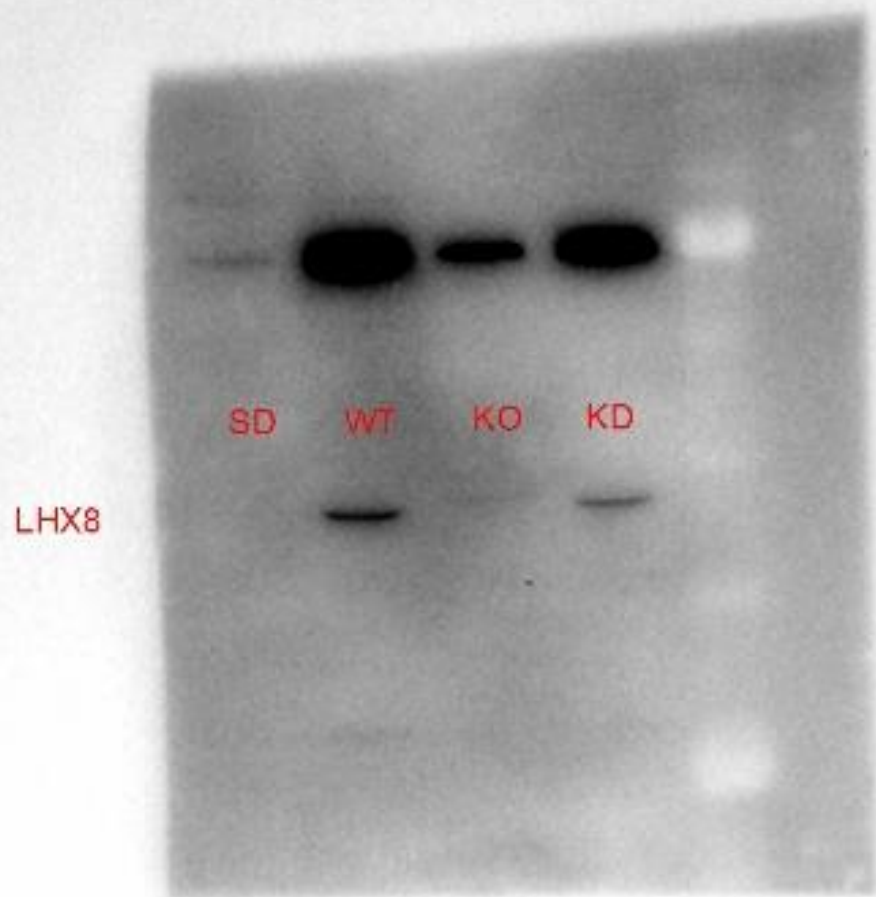

Figure 5C-1

Supplement: Figure 5—source data 1. [file elife-78421-fig5-data1.zip › Figure 5C-source data 1.pdf]

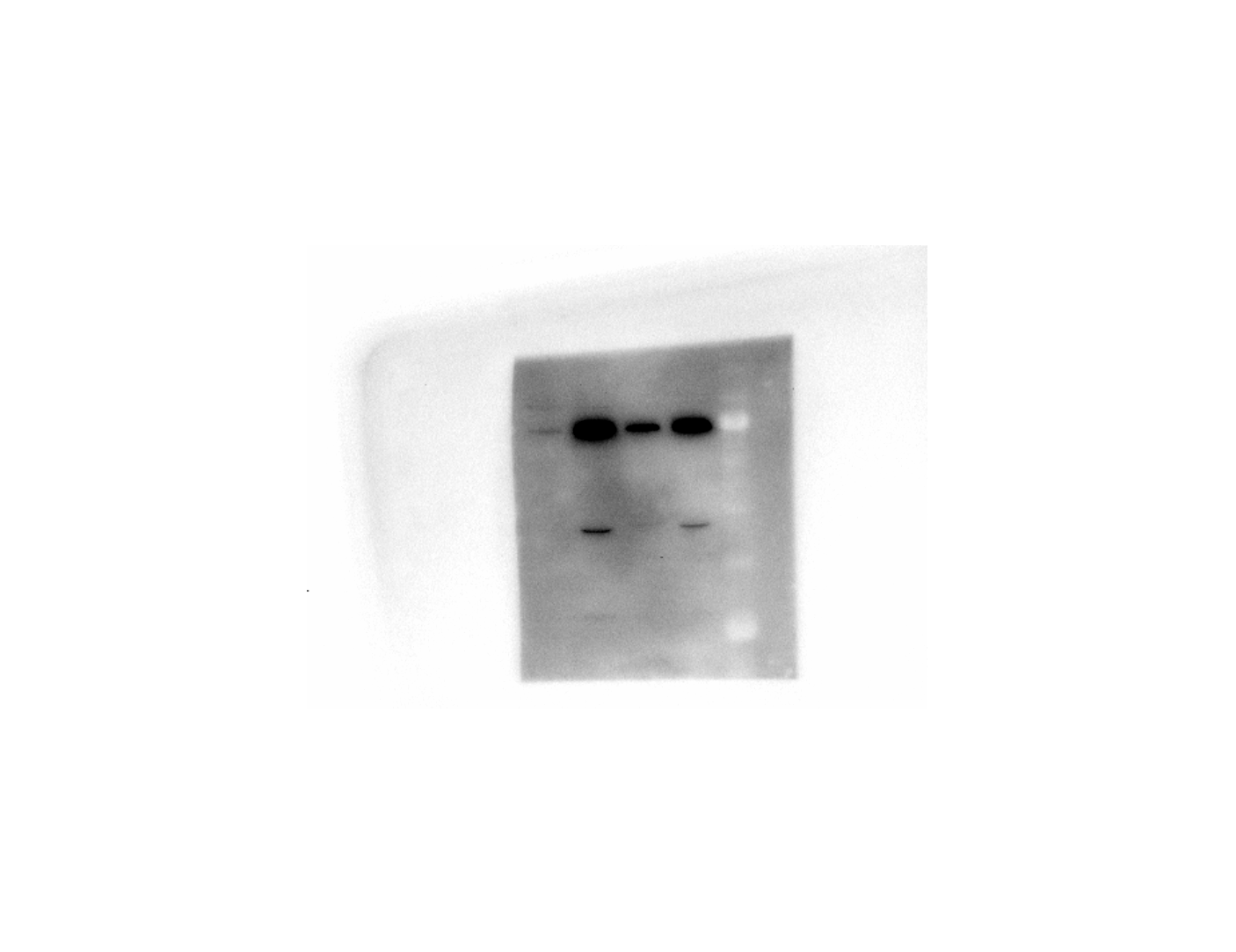

Supplement: Figure 5—source data 1. [file elife-78421-fig5-data1.zip › Figure 5-source data 1-1/Figure 5C-source data 1.tif]

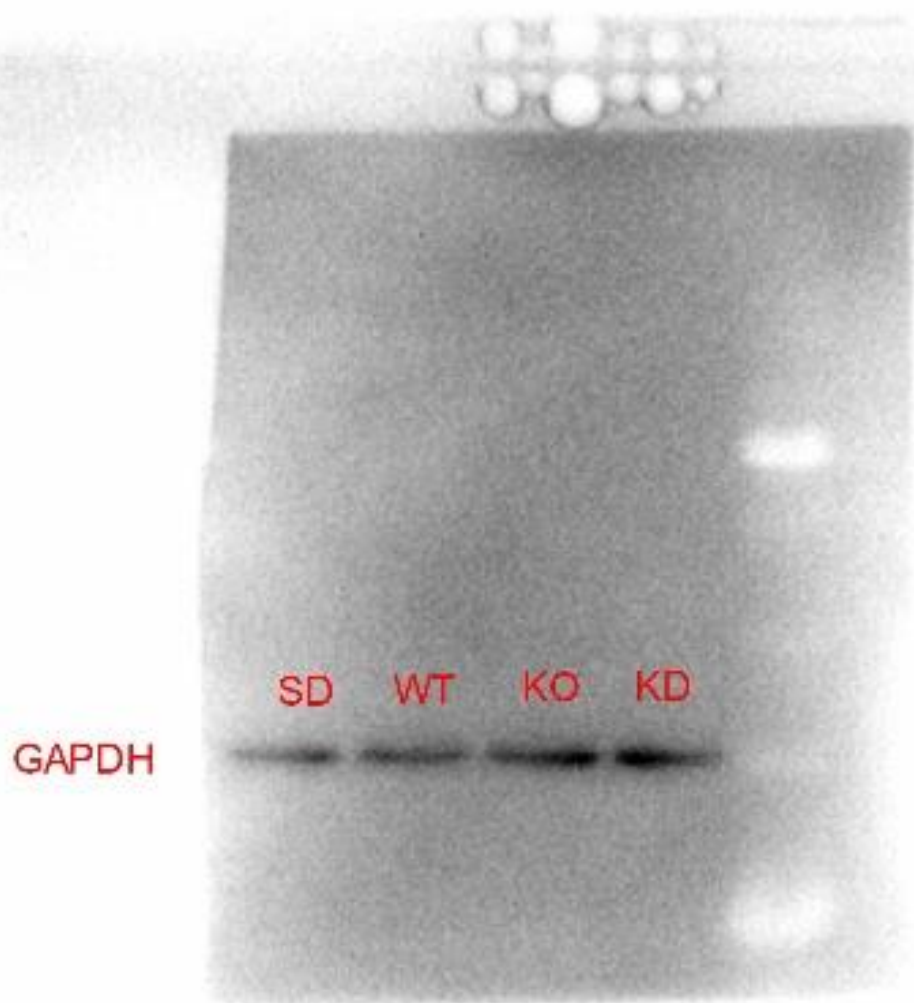

Figure 5C-2

Supplement: Figure 5—source data 2. [file elife-78421-fig5-data2.zip › Figure 5C-source data 2.pdf]

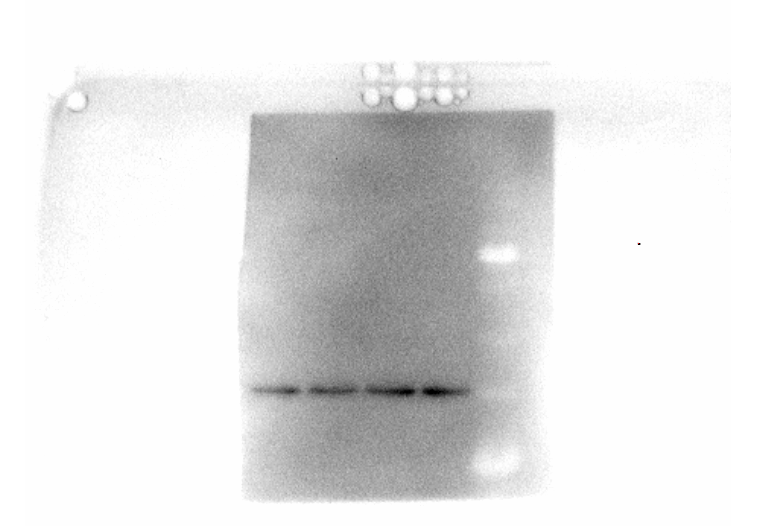

Supplement: Figure 5—source data 2. [file elife-78421-fig5-data2.zip › Figure 5-source data 2-1/Figure 5C-source data 2.tif]

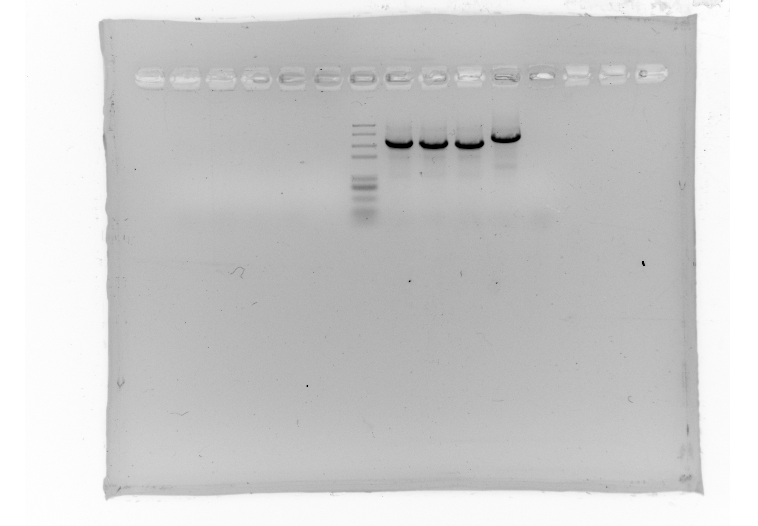

Supplement: Figure 5—figure supplement 1—source data 1. [file elife-78421-fig5-figsupp1-data1.zip › Figure 5-figure supplement 1-source data 1 78421/Figure 5-figure supplement 1A-source data 4.tif]

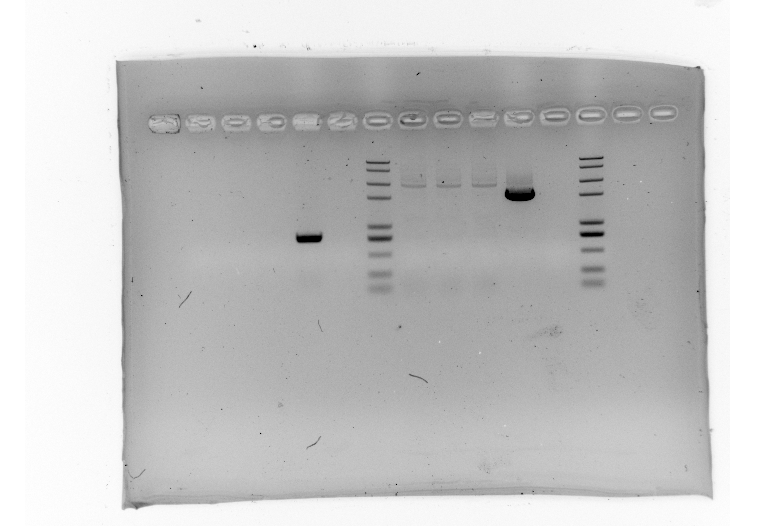

Supplement: Figure 5—figure supplement 1—source data 1. [file elife-78421-fig5-figsupp1-data1.zip › Figure 5-figure supplement 1-source data 1 78421/Figure 5-figure supplement 1A-source data 3.tif]

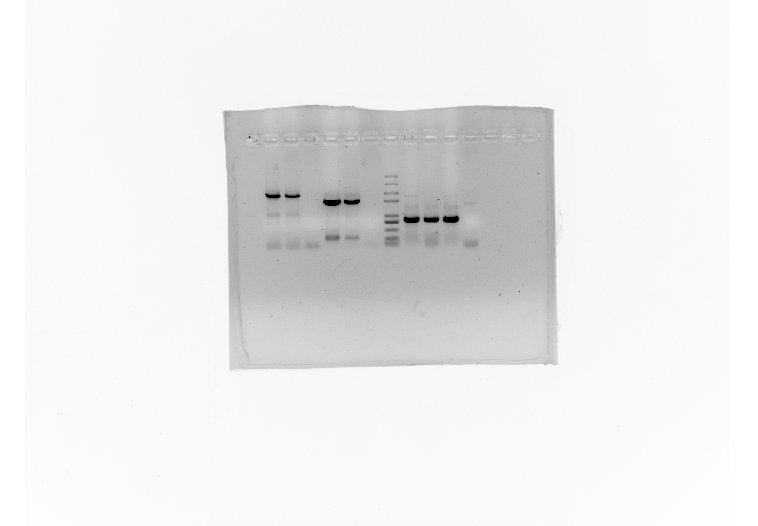

Supplement: Figure 5—figure supplement 1—source data 1. [file elife-78421-fig5-figsupp1-data1.zip › Figure 5-figure supplement 1-source data 1 78421/Figure 5-figure supplement 1A-source data 2.tif]

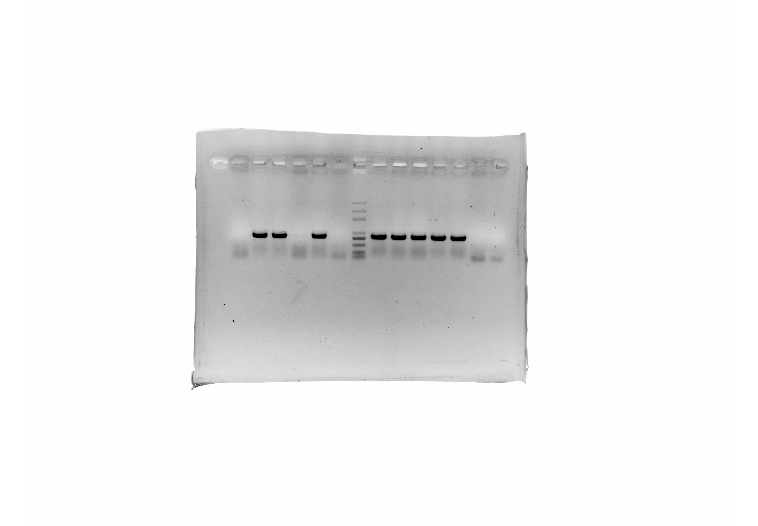

Supplement: Figure 5—figure supplement 1—source data 1. [file elife-78421-fig5-figsupp1-data1.zip › Figure 5-figure supplement 1-source data 1 78421/Figure 5-figure supplement 1A-source data 1.tif]

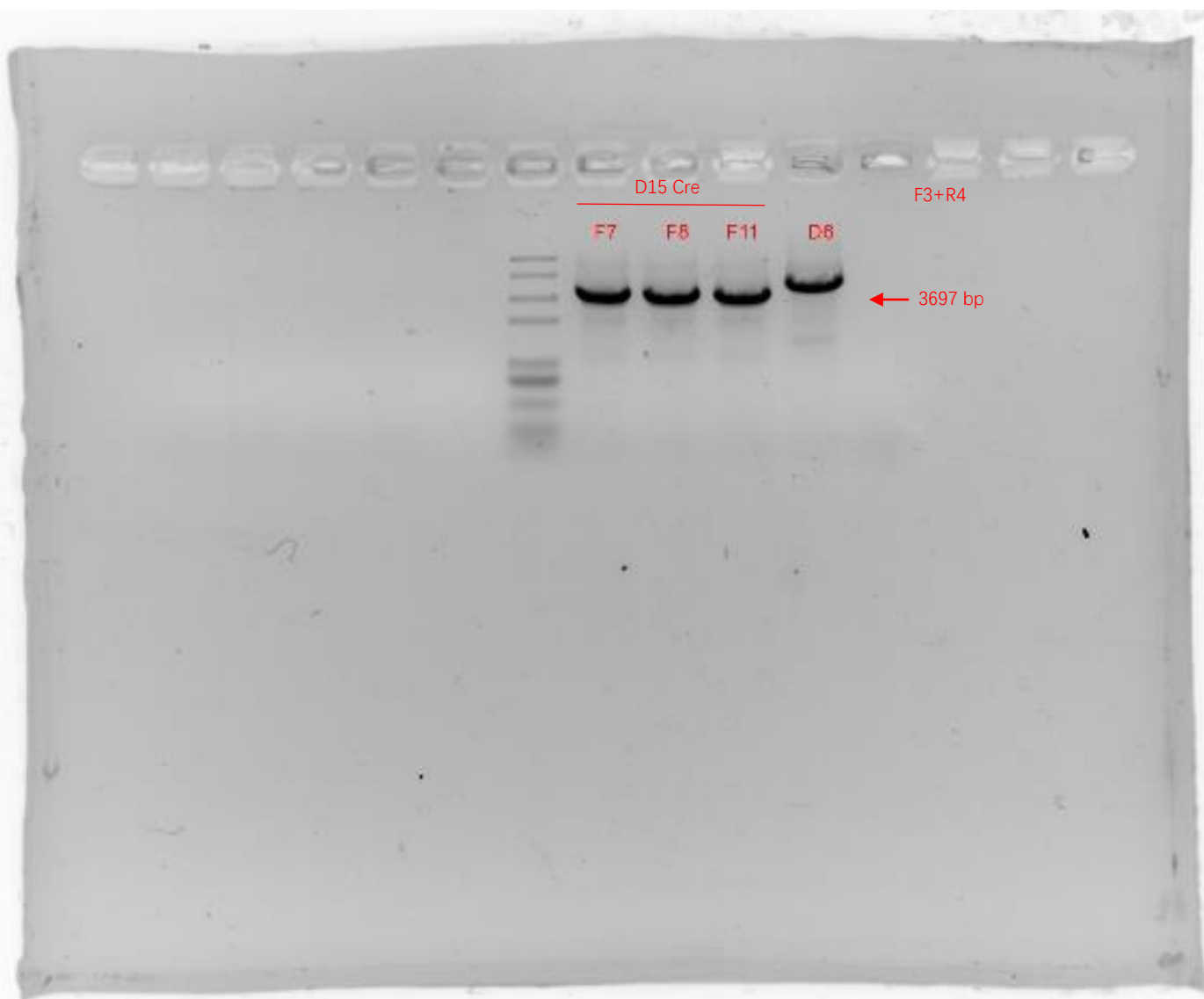

Figure S6A-4

Supplement: Figure 5—figure supplement 1—source data 1. [file elife-78421-fig5-figsupp1-data1.zip › Figure 5-figure supplement 1A-source data 4.pdf]

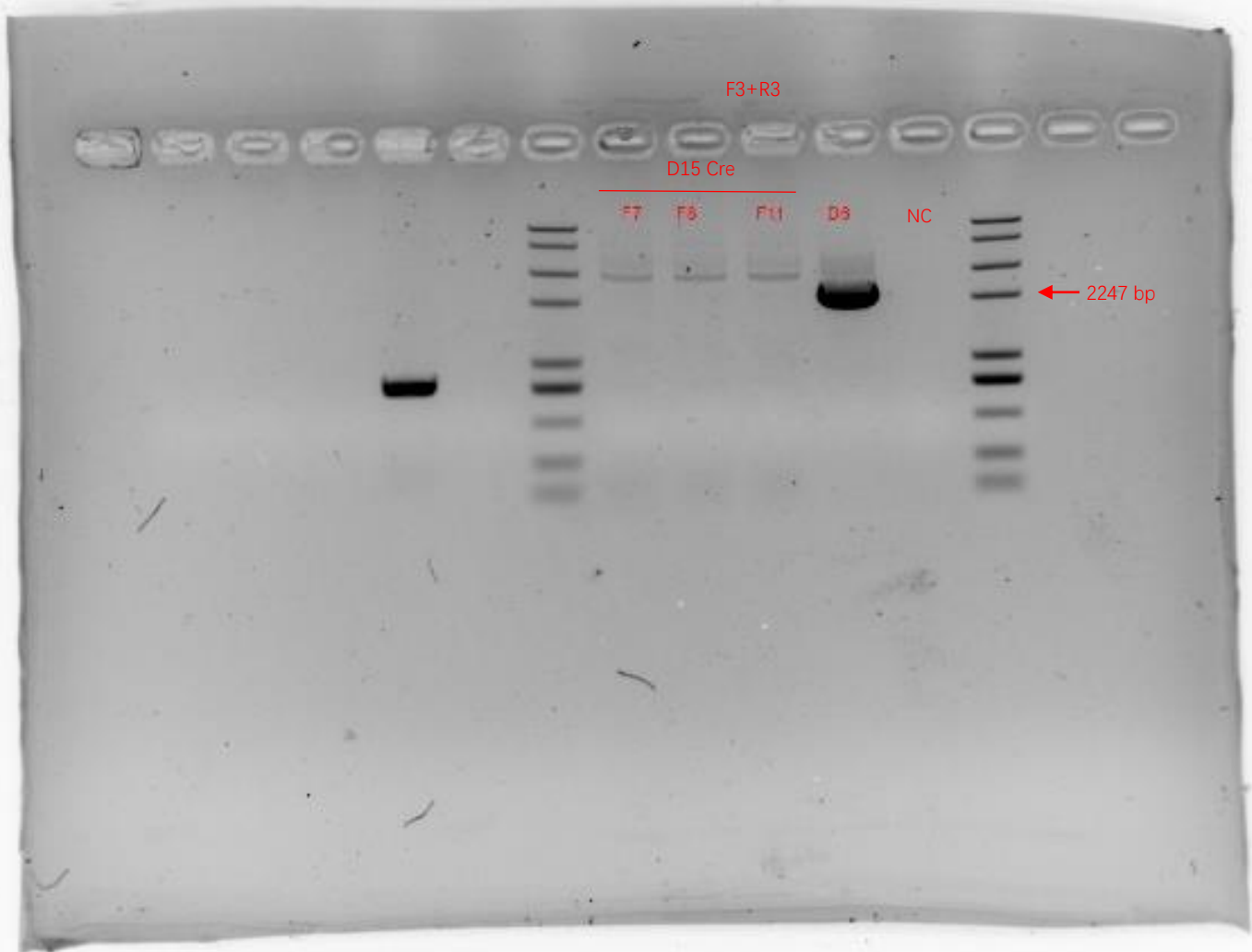

Figure S6A-3

Supplement: Figure 5—figure supplement 1—source data 1. [file elife-78421-fig5-figsupp1-data1.zip › Figure 5-figure supplement 1A-source data 3.pdf]

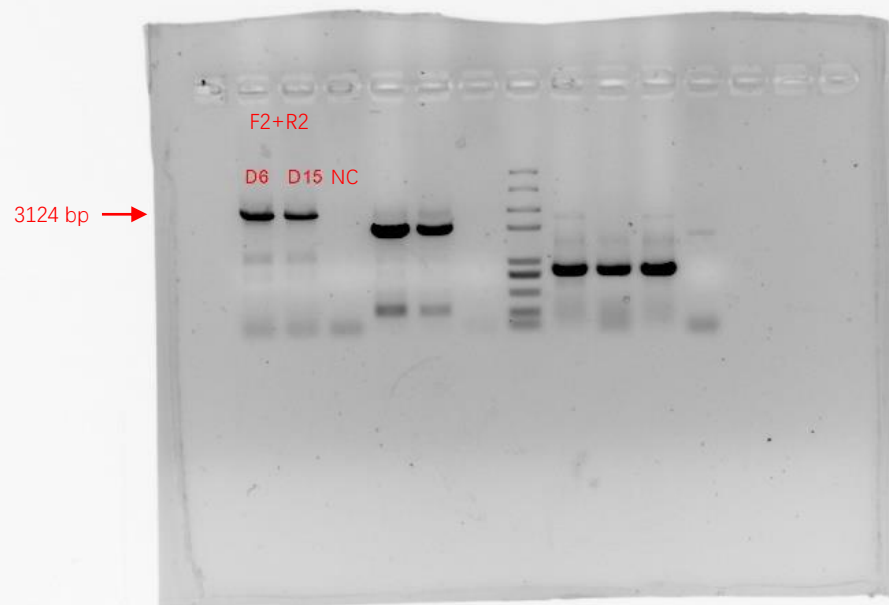

Figure S6A-2

Supplement: Figure 5—figure supplement 1—source data 1. [file elife-78421-fig5-figsupp1-data1.zip › Figure 5-figure supplement 1A-source data 2.pdf]

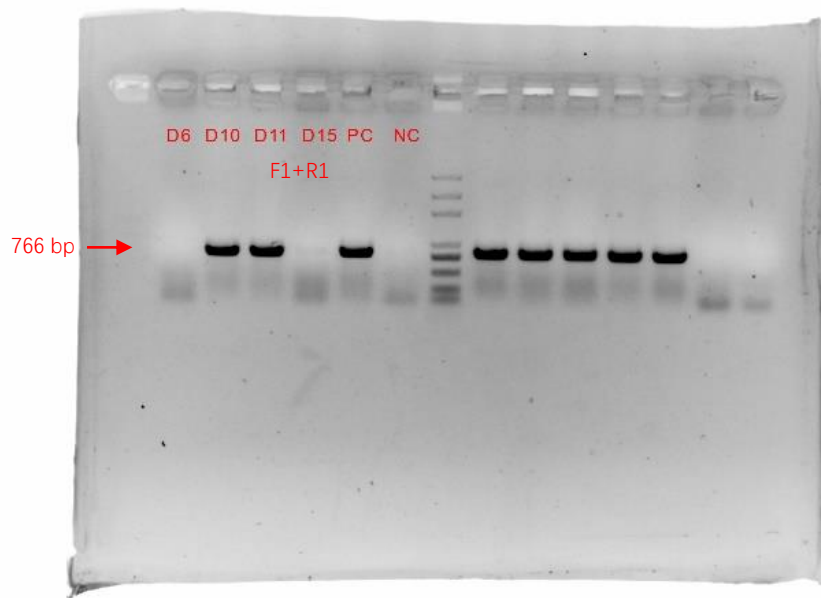

Figure S6A-1

Supplement: Figure 5—figure supplement 1—source data 1. [file elife-78421-fig5-figsupp1-data1.zip › Figure 5-figure supplement 1A-source data 1.pdf]

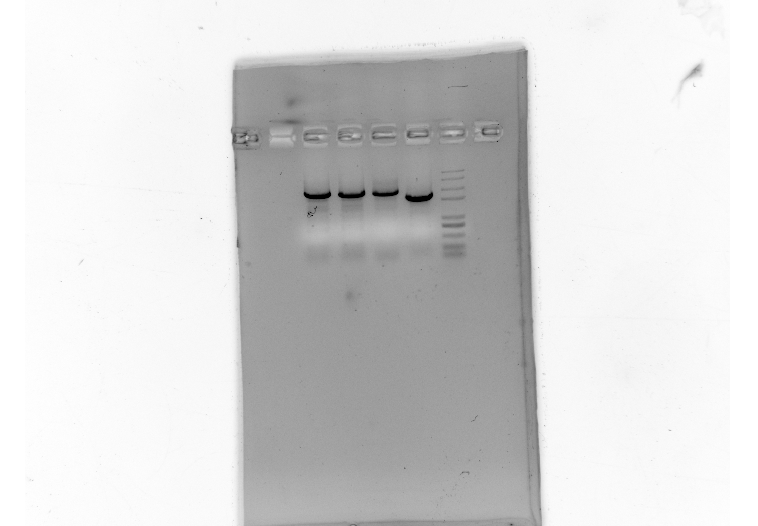

Supplement: Figure 5—figure supplement 1—source data 2. [file elife-78421-fig5-figsupp1-data2.zip › Figure 5-figure supplement 1-source data 2/Figure 5-figure supplement 1E-source data 5.tif]

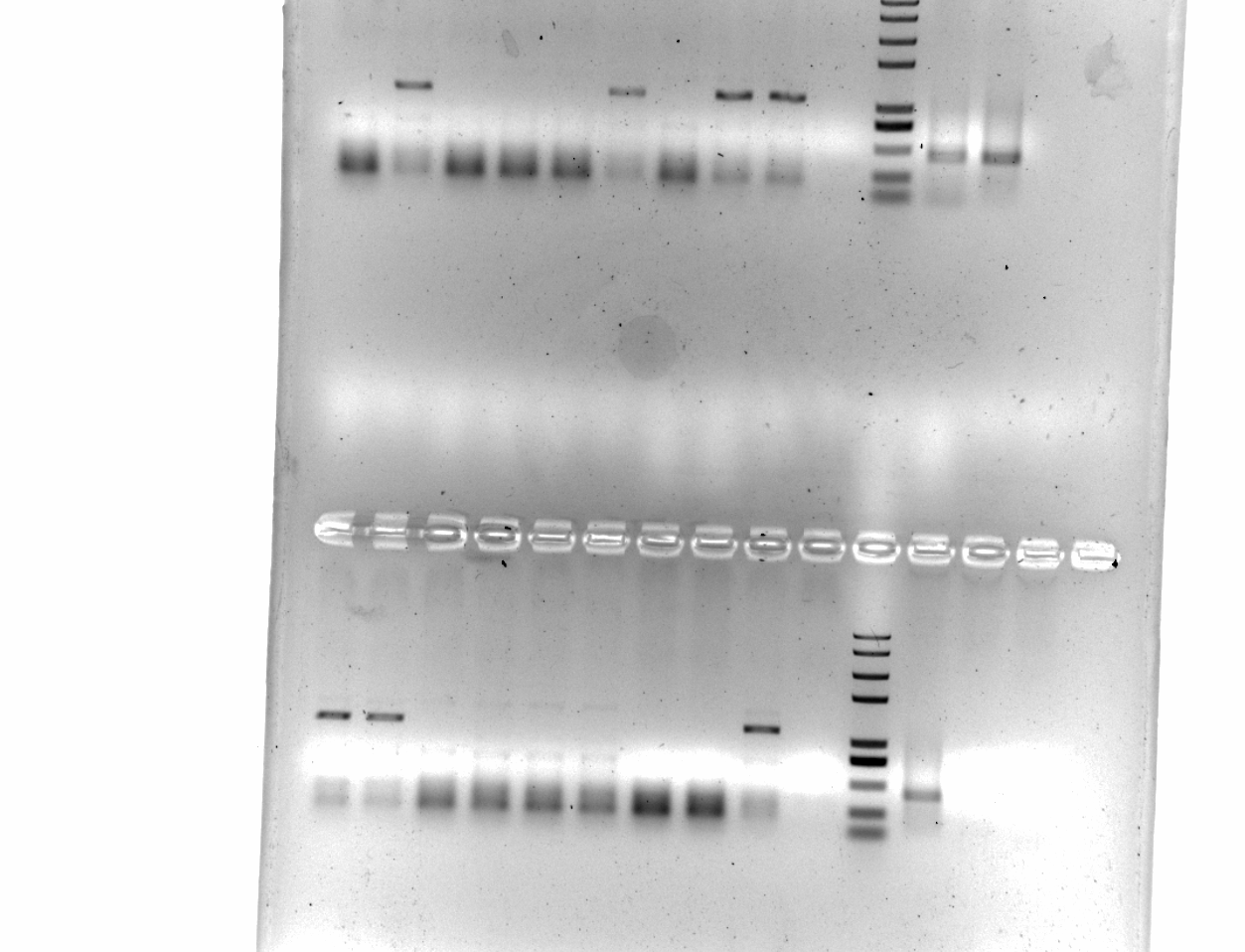

Supplement: Figure 5—figure supplement 1—source data 2. [file elife-78421-fig5-figsupp1-data2.zip › Figure 5-figure supplement 1-source data 2/Figure 5-figure supplement 1E-source data 3 4.tif]

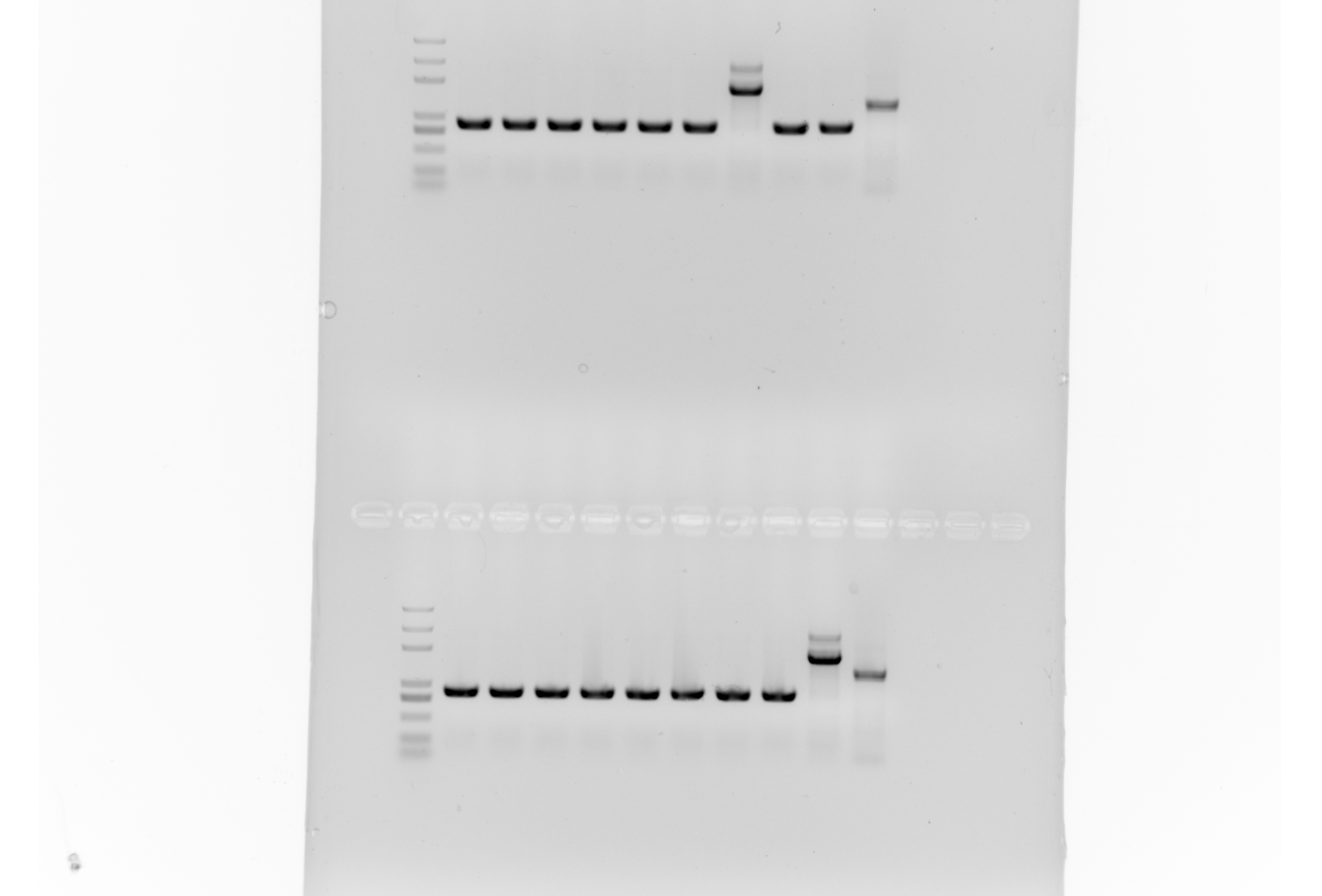

Supplement: Figure 5—figure supplement 1—source data 2. [file elife-78421-fig5-figsupp1-data2.zip › Figure 5-figure supplement 1-source data 2/Figure 5-figure supplement 1E-source data 1 2.tif]

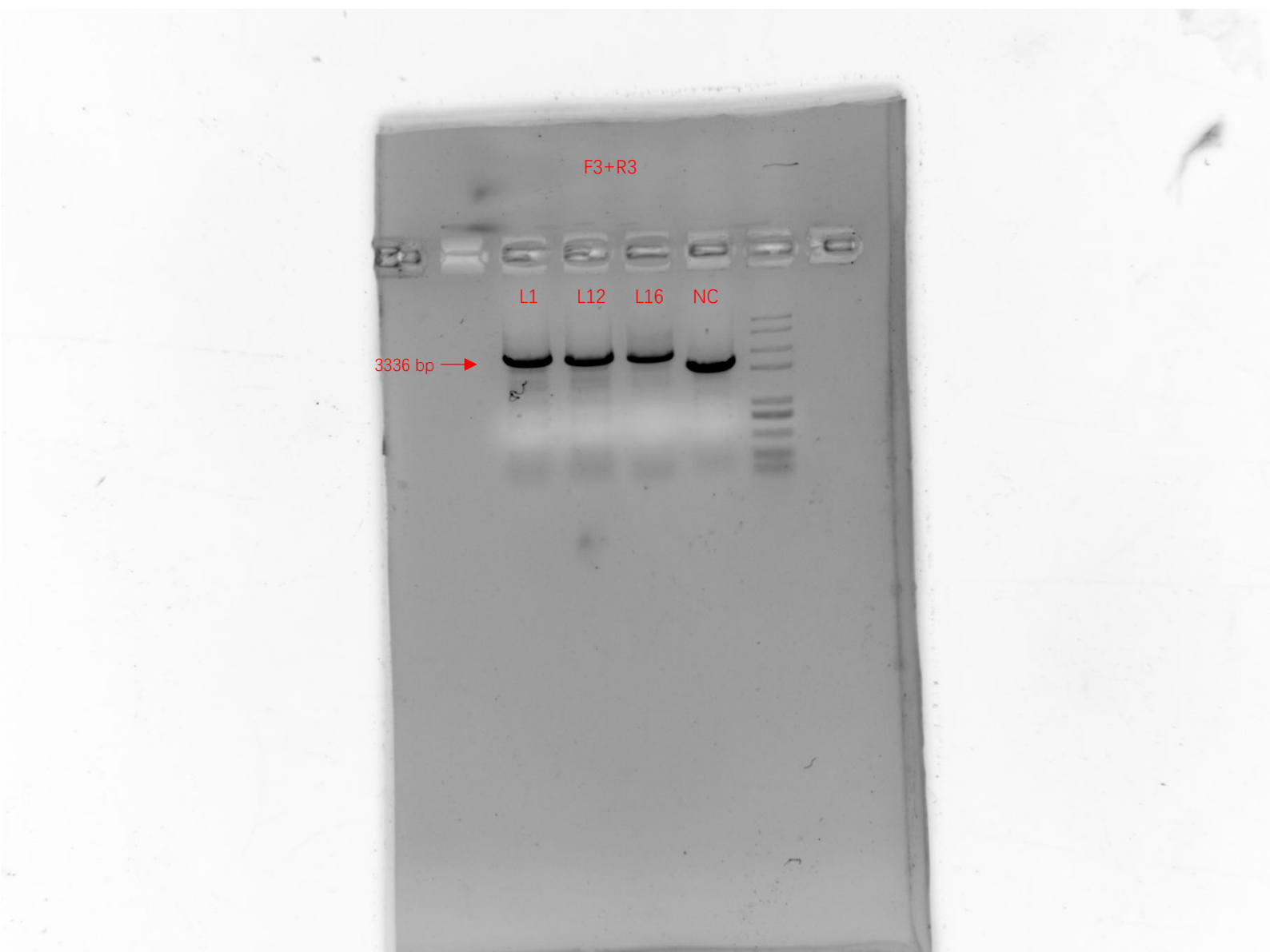

Figure S6E-5

Supplement: Figure 5—figure supplement 1—source data 2. [file elife-78421-fig5-figsupp1-data2.zip › Figure 5-figure supplement 1E-source data 5.pdf]

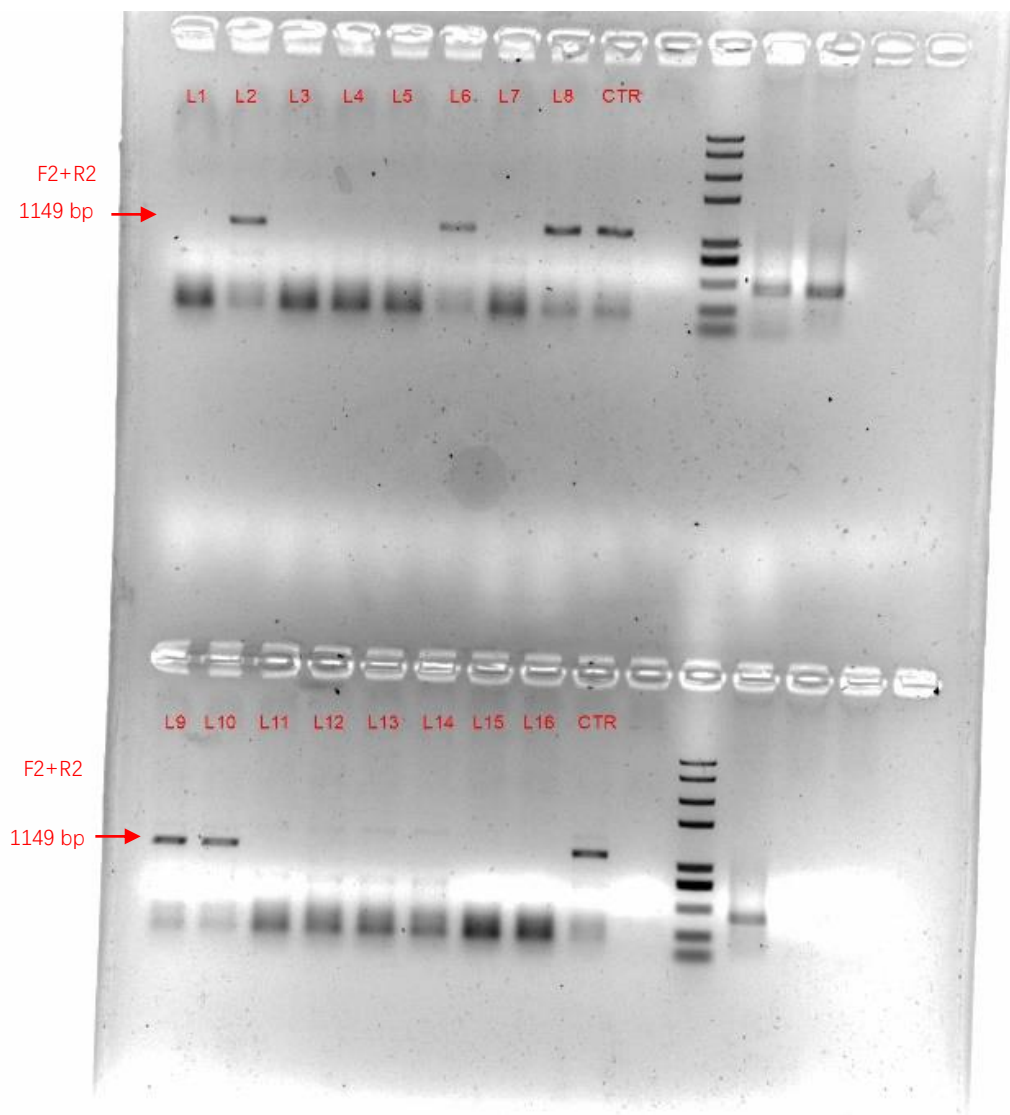

Figure S6E-3 4

Supplement: Figure 5—figure supplement 1—source data 2. [file elife-78421-fig5-figsupp1-data2.zip › Figure 5-figure supplement 1E-source data 3,4.pdf]

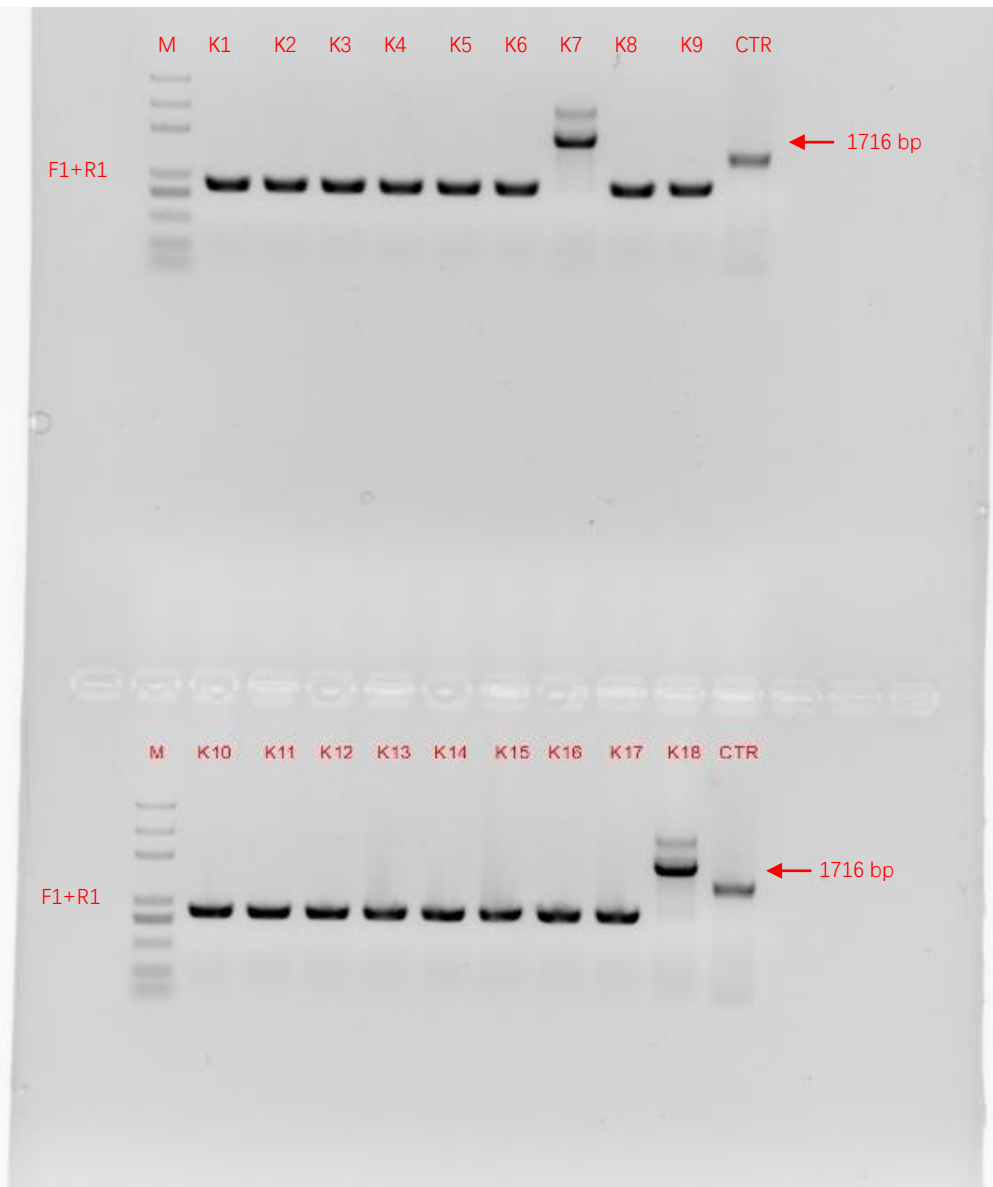

Figure S6E-1.2

Supplement: Figure 5—figure supplement 1—source data 2. [file elife-78421-fig5-figsupp1-data2.zip › Figure 5-figure supplement 1E-source data 1,2.pdf]

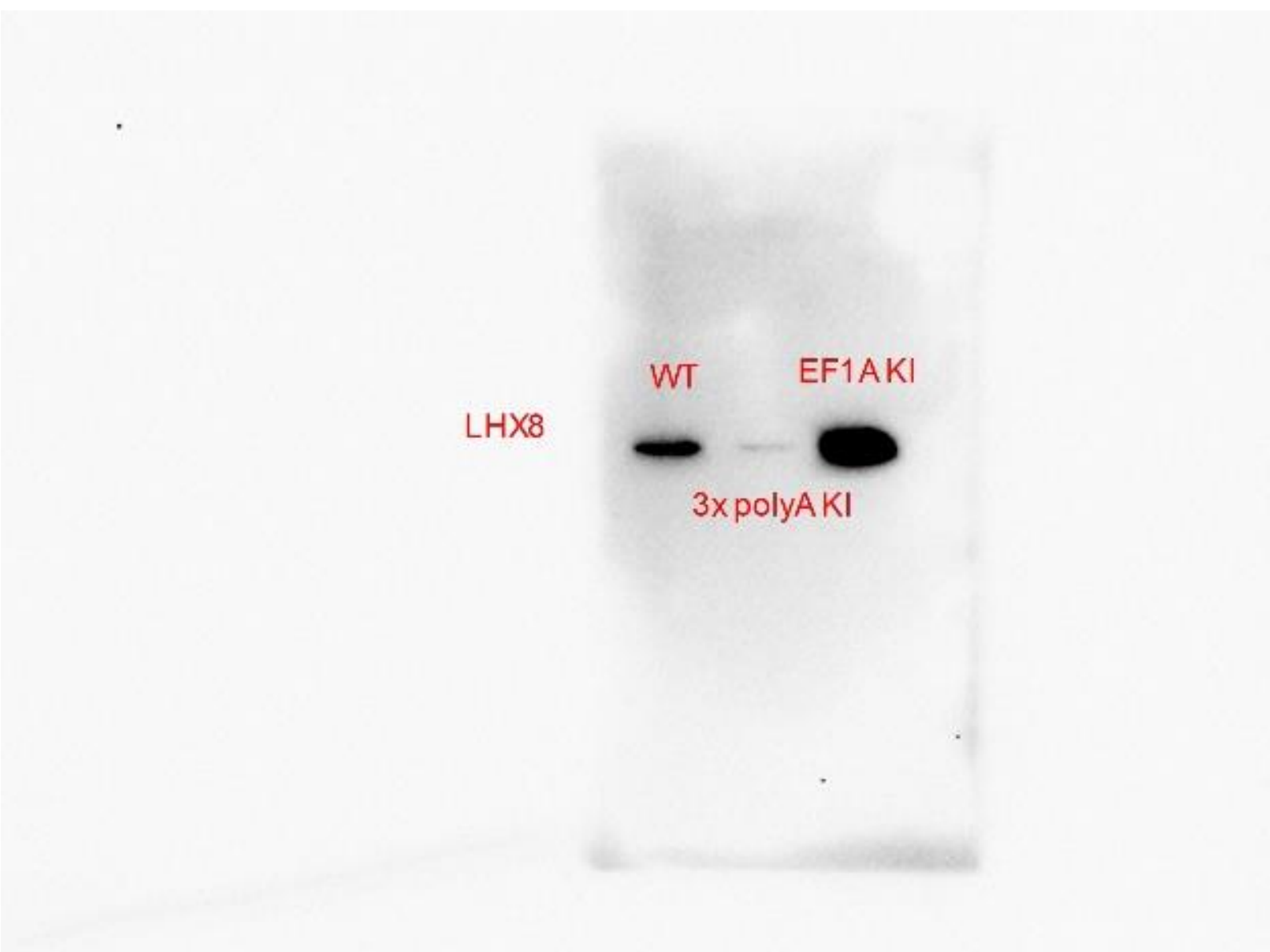

Figure 6C-1

Supplement: Figure 6—source data 1. [file elife-78421-fig6-data1.zip › Figure 6C-source data 1.pdf]

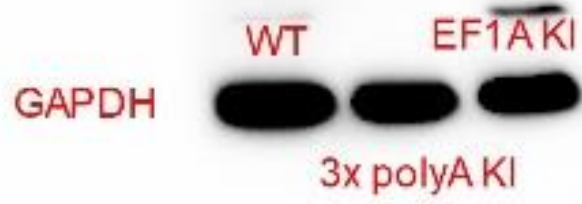

Figure 6C-2

Supplement: Figure 6—source data 2. [file elife-78421-fig6-data2.zip › Figure 6C-source data 2.pdf]

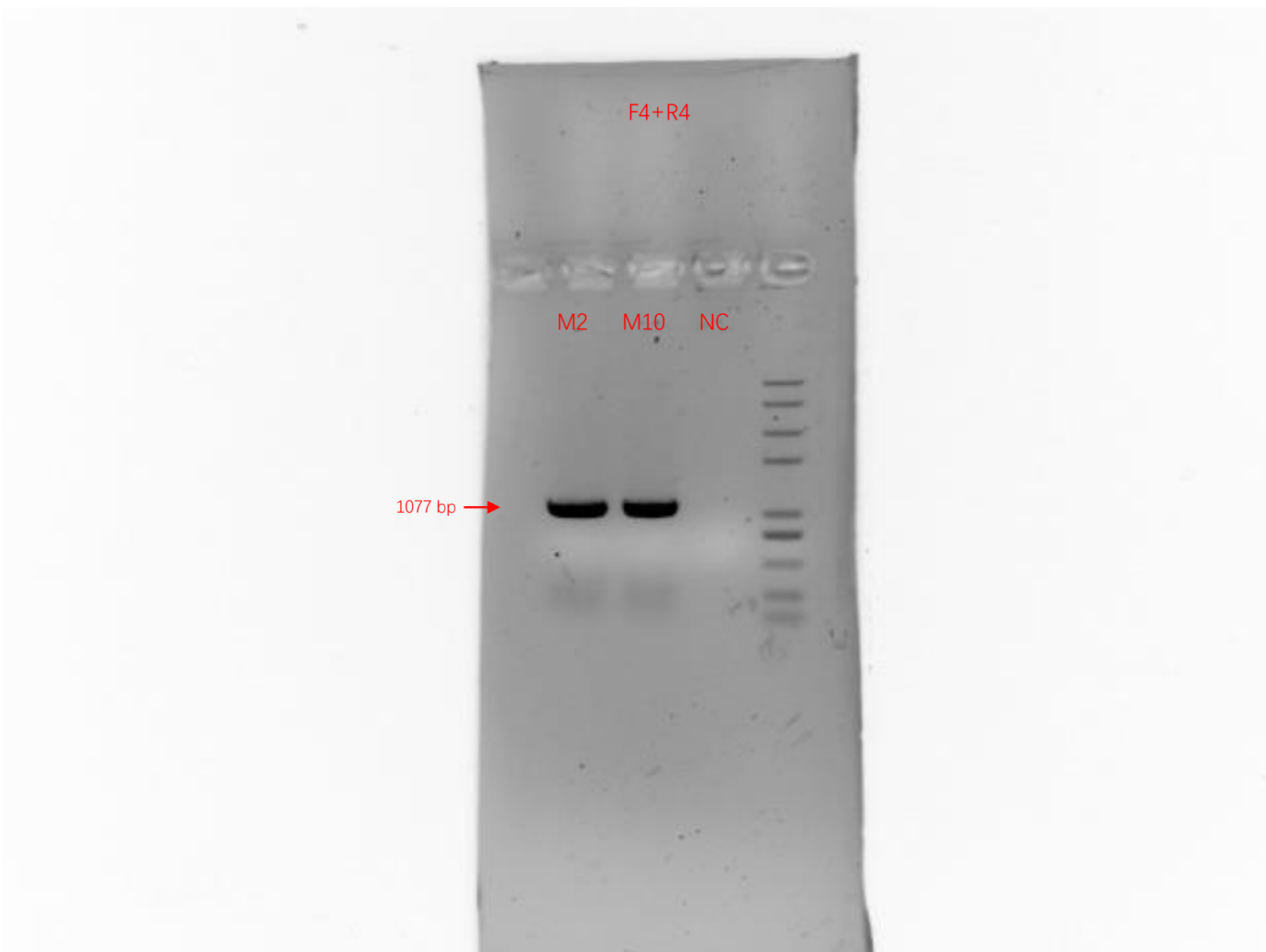

Figure S7A-4

Supplement: Figure 6—figure supplement 1—source data 1. [file elife-78421-fig6-figsupp1-data1.zip › Figure 6-figure supplement 1A-source data 4.pdf]

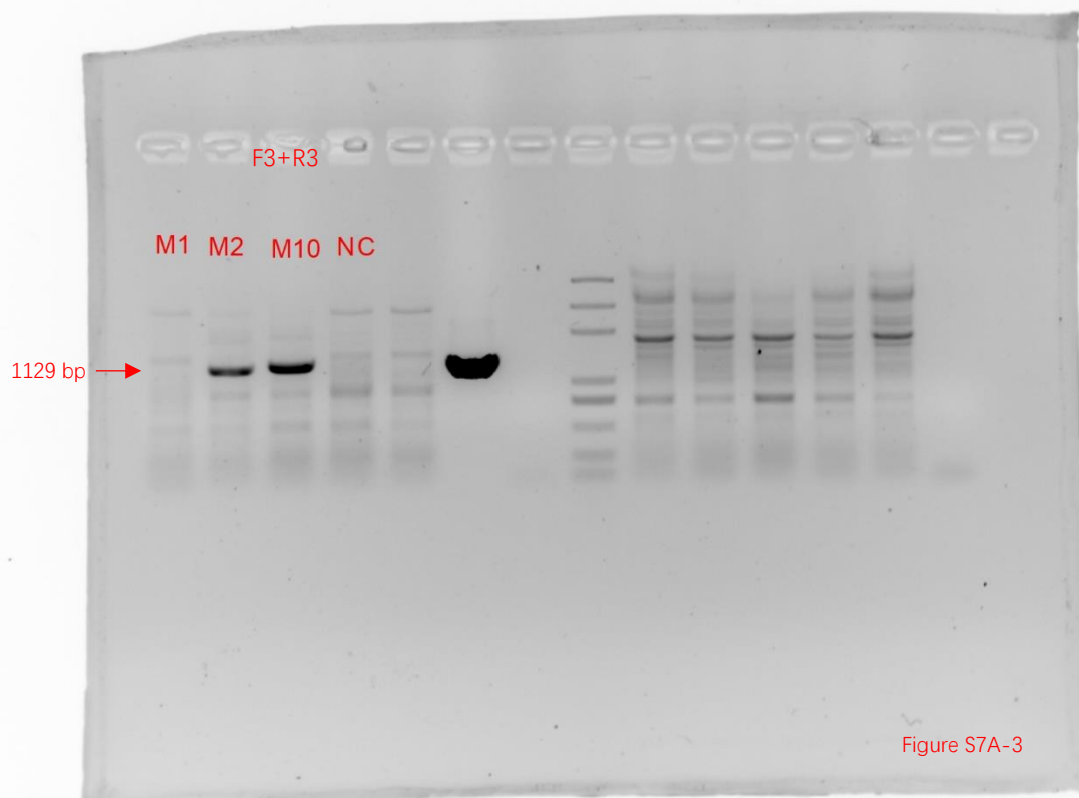

Figure S7A-3

Supplement: Figure 6—figure supplement 1—source data 1. [file elife-78421-fig6-figsupp1-data1.zip › Figure 6-figure supplement 1A-source data 3.pdf]

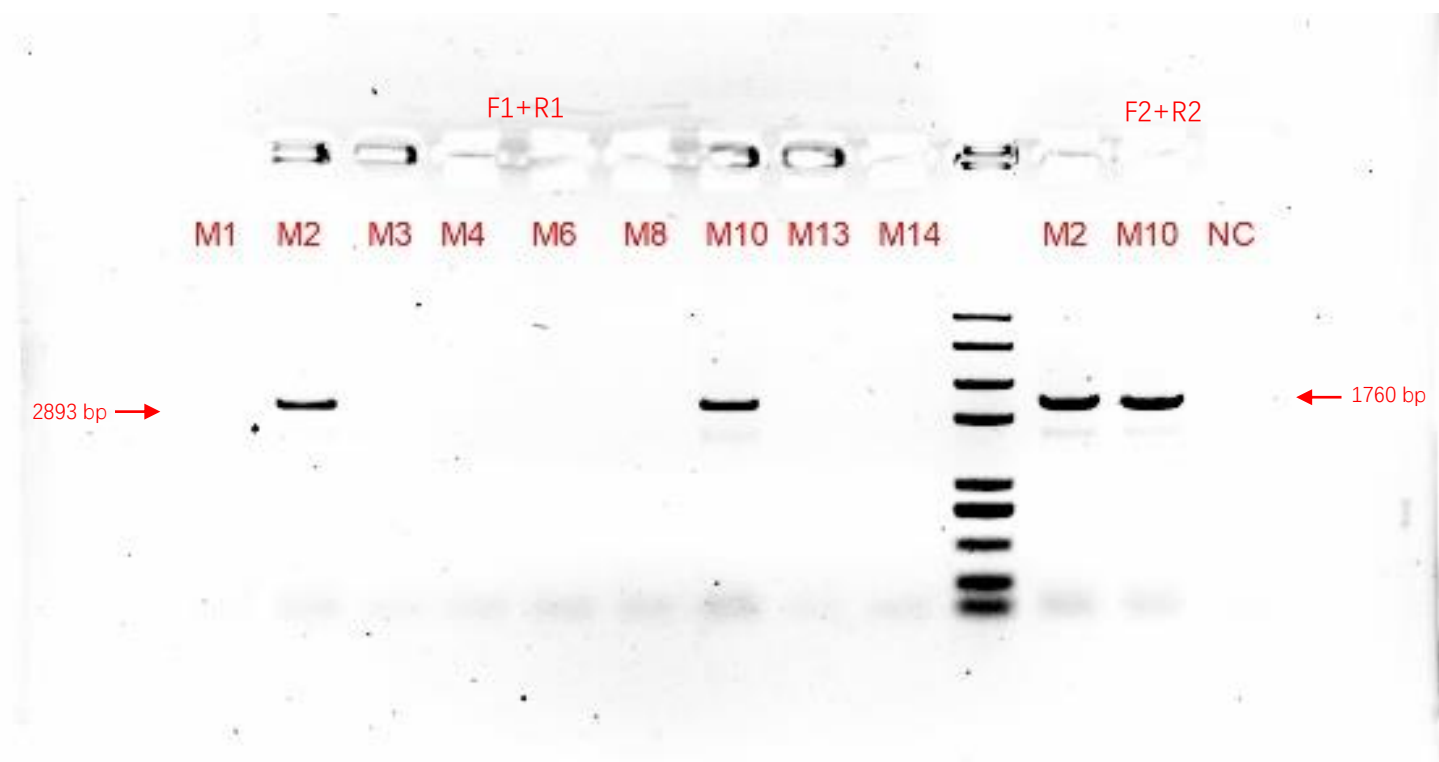

Figure S7A-1 2

Supplement: Figure 6—figure supplement 1—source data 1. [file elife-78421-fig6-figsupp1-data1.zip › Figure 6-figure supplement 1A-source data 1,2.pdf]

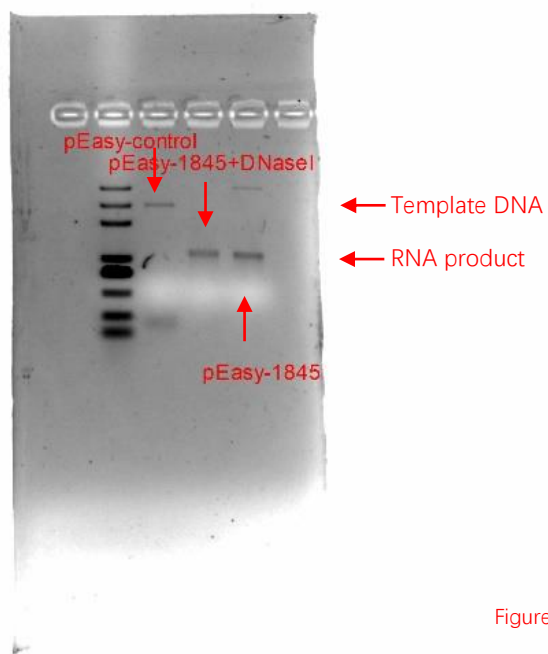

Figure S7D

Supplement: Figure 6—figure supplement 1—source data 2. [file elife-78421-fig6-figsupp1-data2.zip › Figure 6-figure supplement 1D-source data.pdf]

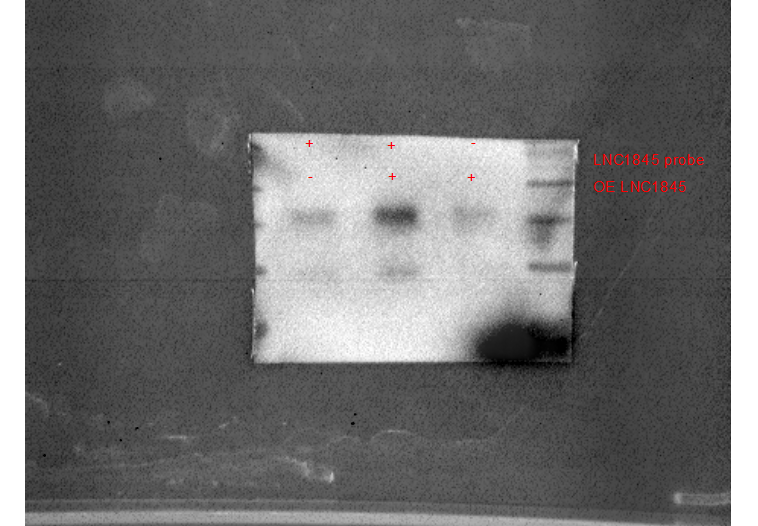

Supplement: Figure 6—figure supplement 2—source data 1. [file elife-78421-fig6-figsupp2-data1.zip › Figure 6-figure supplement 2-source data 1-3/labelled Figure 6-figure supplement 2-source data 1.tif]

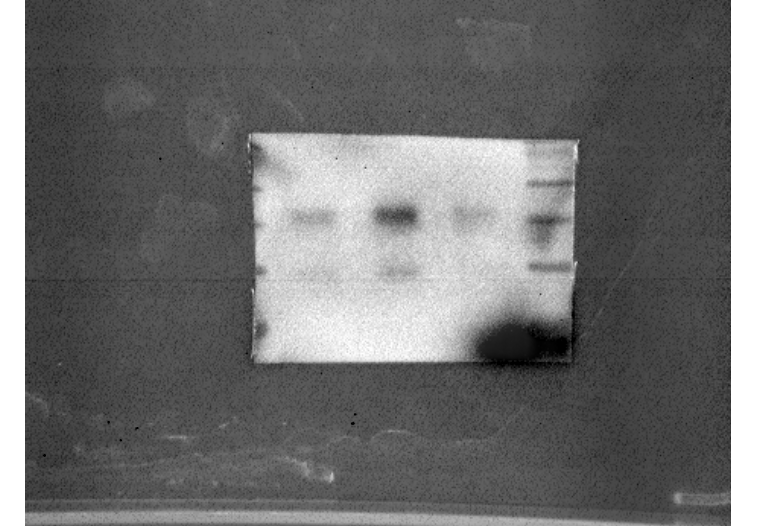

Supplement: Figure 6—figure supplement 2—source data 1. [file elife-78421-fig6-figsupp2-data1.zip › Figure 6-figure supplement 2-source data 1-3/Figure 6-figure supplement 2-source data 1.tif]
